# 

**Published:** 1887-12

**Authors:** 


CONTENTS:

ARTICLE I.—Southern Dental Association.......................................... 337

" II.—American Dental Society of Europe .......... . ....... .... 360

" III.—Soft Gold Foil....... ............ ..... .............................. 375

EDITORIAL, ETC.

Experience of an English Dentist who had Two Teeth Implanted...... 382

Examination Questions—Royal College of Surgeons of England .. ... 384

